# Lifestyle, behavior, perception and practices of Nepalese during lockdown due to COVID-19 pandemic

**DOI:** 10.31729/jnma.5284

**Published:** 2020-09-30

**Authors:** Samikshya Kandel, Mahesh Lamsal, Saroj Adhikari Yadav, Dipak Bhandari, Ganesh Adhikari, Sagar Poudel, Pawan Sharma, Swotantra Gautam

**Affiliations:** 1 Nepal Academy of Science and Technology, Khumaltar, Lalitpur, Nepal; 2 Central Department of Biotechnology, Tribhuvan University, Kirtipur, Kathmandu, Nepal; 3 Patan Academy of Health Sciences, Lalitpur, Kathmandu, Nepal; 4 Pokhara Biological Sciences and Technology Centre, Pokhara, Nepal; 5 All India Institute of Medical Sciences, New Delhi, India; 6 B.P. Koirala Institute of Health Sciences, Dharan, Nepal

**Keywords:** *COVID-19*, *lockdown*, *Nepal*, *Pandemic*

## Abstract

**Introduction:**

COVID-19 infection is caused by a new strain of SARS CoV-2 virus, which transmits directly from person-to-person and has become a pandemic. To counteract this, actions related to mass quarantines or stay-at-home orders have been used termed as lockdown. This study aims to study lifestyle, behaviour, perception and practice of people regarding during the lockdown.

**Methods:**

An online survey was conducted with structured questionnaire in Google forms after ethical approval from Nepal Health Research Council (Ref-2631). The attributes of knowledge, attitude and practices were explored using multiple-choice questions and results were statistically analysed using Microsoft excel.

**Results:**

Five hundred fifty-five respondents completed the survey with 280 (50.5%) males and 275 (49.5%) female. The knowledge regarding viral pandemic was increased in 496 (89.3%) respondents. 424 (76.4%) people developed stress due to pandemic. Three hundred fifty three (63.6%) were adversely affected by professional works or suffered economic loss in business. More than 42% participants are using their time for study in personal development, online classes etc.

**Conclusions:**

The knowledge of viral pandemic as well as personal hygiene habits have improved in majority of people but many also developed stress. They were convinced that lockdown lowered transmission of infection which in turn affected lifestyle behaviour and practices. Practicing social distancing becomes too difficult for the poor in the absence of proper social security system and government support. E-Learning has become more acceptable due to lockdown. Further studies with in-person interviews are warranted.

## INTRODUCTION

Coronavirus disease 2019 (COVID-19) is caused by Severe Acute Respiratory Syndrome Coronavirus 2 (SARS-CoV-2).^1^ COVID-19 is the third corona-virus outbreak in last 20 years after SARS-CoV and MERS-CoV.^2^ This is highly infectious with main clinical features like fever, cough, fatigue, anorexia, shortness of breath, sputum production and myalgia.^3^ The first case was reported in Wuhan, Hubei province of China at the end of 2019. It has rapidly spread, resulting in an epidemic throughout China, followed by an increasing number of cases throughout the world.^4^ World health organization (WHO) declared a public health emergency of international concern on 30 January 2020.^5^ The COVID-19 was declared a pandemic on 11 March 2020.^6^

There are over 9.6 million infected cases and mortality over 491 thousand worldwide by June 27.^7^ To prevent the virus from spreading, initially Chinese government put up the “lockdown” measure in Wuhan on 23 January 2020, which was a new approach to control infectious diseases by mass-quarantine or stay-at-home orders.^8,9^ Then othercountries have followed the same.^9^ In Nepal, country-wide lockdown came into effect from 24 March to 14 June, 2020.^10^ Due to lockdown people are compelled to spend their time at home. Most of the people throughout the country are directly affected by it. There are changes in the health-related behaviour during the lockdown period. The knowledge, attitude and behaviour among the general population can define the direction that a pandemic will take. There are very few literatures published regarding this area during current COVID-19 pandemic.

Our study aims to explore the lifestyle behaviour, perception and practice of people during this lockdown.

## METHODS

This descriptive cross-sectional study was conducted in May 2020. An online survey using a cross-sectional design and convenient sampling was conducted. The online data collection methodology was chosen due to its benefits like ease of access, cost efficiency, and its usefulness and practical advantages. A link of the survey was sent to the friends and relatives of researchers and snow-balling was done. Also the survey form was posted in online social media. Those people willing to participate and provide written informed consent (online) were enrolled in this study. Those not responding within 1 week were excluded. Most of the participants were colleagues, friends and family of authors residing in Nepal and different parts of the world. A total of 26 questions were included in the questionnaire. The questionnaire consisted of different sections including demographics, knowledge for specific fields and daily health practices of people participating in the research. The questions were finalized on the basis of face validity and consensus among the researchers. Demographic variables included were age, sex, education status, occupation, and different geographical locations. Behavioural variables included were hand washing habits, use of preventive measures, development of stress, level of knowledge and information regarding viral pandemics and so on.

Ethical approval for the research was taken from Nepal Health Research Council (NHRC). Participants who gave consent to willingly participate in the survey clicked the Continue button and responded to the questionnaire. Those who did not consent were automatically excluded. All the data related to personal details of participants are kept confidential.

The data entry and analysis were done in Microsoft excel. At first, pretesting was done among 10 people; the findings and suggestions were carefully discussed, though no significant changes were needed in questionnaire. Then study was conducted. Descriptive statistics were used to describe the characteristics of the sample and knowledge, attitude and practices of the participants towards COVID-19.

## RESULTS

Total number of 555 participants filled up the online questionnaire survey. The socio-demographic characteristics are shown (Table 1). The male to female ratio of our respondents was 1.02 with male 280 (50.5%) and female 275 (49.5%). Majority of respondents were of age 20-29 years and were educated. Most of the participants in this research, 507 (91.4%) were residing in Nepal and only 48 (8.6%) were from outside Nepal.

**Table 1:** Demographic Characters of participants involved in the survey.

Characteristics		n (%)
Gender	Male	280 (50.5%)
	Female	275 (49.5%)
Age (in years)	Below 20	30 (5.4%)
	20-29	457 (82.3%)
	30-39	53 (9.5%)
	40-49	8 (1.5%)
	Above 50	7 (1.3%)
Education level	High School	45 (8.1%)
	Bachelors	279 (50.3%)
	Masters and above	231 (41.6%)
Current Location	Nepal	507 (91.4%)
	Outside Nepal	48 (8.6%)

To find about perception, experience, knowledge, understanding, and lifestyle patterns of the respondents during COVID-19 pandemic, a number of questions were included as shown (Table 2). Majority of the participants, 530 (95.5%) responded that they were residing in lockdown area for an average period of 51±12days. Majority of respondent mentioned that their understanding of viral infection and transmission had increased due to awareness created in the society by different mediums during this pandemic. A large number of respondents 329 (59.3%) thought that the risk of infection had decreased by lockdown, while 57 (10.3%) think it didn't.

**Table 2:** Perceptions and lifestyle behavior of participants towards COVID-19 pandemics.

Questions	Responses		
	Yes	No	May Be
Are/were you in lockdown?	530 (95.5%)	25 (4.5%)	-
Do you think, the risk of COVID-19 pandemic, has been lowered by lockdown strategy?	329 (59.3%)	57 (10.3%)	169 (30.4%)
Do you think, the level of your knowledge and information regarding viral infection and epidemics has been widened due to this pandemic?	496 (89.3%)	48 (8.6%)	11 (1.9%)
Do you think, your personal hygiene habits have improved due to awareness during COVID-19 pandemic?	498 (89.8%)	46 (8.3%)	11 (1.9%)
Have you ever tried violating the rules from your authorities?	63 (11.4%)	481 (86.7%)	11 (1.9%)
Did you use preventive measures like mask, sanitizer gloves to prevent COVID-19 transmission?	539 (97.1 %)	16 (2.8%)	
Were you stressed during COVID-19 pandemic?	424 (76.4%)	111 (20%)	20 (3.6%)

The improvement of personal hygiene practice was found to have increased in 498 (91.5%) people due to the awareness programs launched during COVID-19 pandemic. Regarding the usage of the preventive measures like mask, sanitizer, gloves and hand-wash, majority were using protective/preventive measures, however, 16 (2.8%) were still devoid of this. Many respondents were found re-using the same mask as well. Regarding hand-washing practice: most of the participants answered that they preferred washing their hands with soap-water rather than plain water while 37 (6.7%) respondents preferred using sanitizer only. Some of the participants, 271 (48.8%) used both soap water and sanitizer. Sixty-two (11%) of participants used only plain water to wash their hands.

Alarmingly, 424 (76.4%) respondents accepted being stressed during lockdown period. Supply of goods was hard for 143 (25.8%) of them, and, daily requirement supply was not available at all for 21(3.8%) of the total respondents (Table 3). Response from family members to participants on returning to their house, who had been outside due to several reasons during the lockdown period was asked in which 221 (39.8%) received a normal response as before, while 46 (8.3%) were taken skeptically and 83 (14.9 %) were kept a distance apart. Only 38 (6.8%) were quarantined after their return to their home and 13 (2.3%) didn't want to respond to this question.

**Table 3:** Attitude and practices of participants towards study variables.

Questions	Attributes	n (%)
How easily are the daily requirements (like food, medicine, water etc.) available during lockdown?	Easily	367 (66.1%)
	With Hardship	143 (25.8%)
	Not available at all	21 (3.8%)
	No response	24 (4.3%)
How frequently do you change mask?	Use it only once	202 (36.4%)
	Reuse as much as I wish it	99 (17.8%)
	Reuse after washing	214 (38.6%)
	Don’t Use	29 (5.3%)
	Did not respond	11 (1.9%)

In this survey, 238 (42.9%) people were found to utilize their time for study, online classes and personal works while 169 (30.5%) found a positive impact personally and utilizing the lockdown period for learning and developing skills. On the other hand, 15 (2.7%) responded to be bored due to lockdown. In turn, 353 (63.6%) reported to be adversely affected professionally, suffered economic loss in business, or lost their job during lockdown period (Table 4 and 5). This survey also showed that most of the people abided by the rules and regulations of lockdown strategy but 11.6% violated them. Also, 19 (3.4%) were punished for the commit and same fact and neither were they caught violating the lockdown.

**Table 4:** Participant’s response to how their time has been during lockdown days.

Ways of time spend during lockdown	n (%)
Enjoyed (Amazing family time, meeting relatives etc.)	23 (1.4%)
Utilized time (work, study, online class, personal development skills etc.)	238 (42.9%)
Bored	15 (2.7%)
Served in social camp	40 (7.2%)
Not responded	25 (4.5%)
Selected multiple choices	214 (38.6%)

**Table 5:** Effects on population included in the survey due to COVID-19 lockdown.

Impacts of lockdown	n (%)
Economic loss, Lost job, Study/exam/Health checkup etc. affected	353 (63.6%)
Suitable self-time (utilized for personal works and development)	169 (30.5%)
Others	20 (3.6%)
No response	13 (2.3%)

When the respondents were asked about their opinion regarding further management of pandemic: majority of the people 364 (65.6%) emphasized focusing on enhancement of the testing to control the infection while 54 (9.7%) respondents wanted strategy of lockdown to be changed. Only 36 (6.5%) respondent was okay with the lockdown to be continued.

Majority of the participants responded that the questions provided in the survey were relevant enough to address the burning concerns of people. As much as 143 (25.7%) of people found it wonderful filling up the form and 103 (18.6%) of the responses suggest that the respondents felt connected with it.

## DISCUSSION

Our study highlights, respondents were convinced that lockdown decreases infection rate of COVID-19. A study done during this pandemic showed that lockdown was effective in delaying the spread of COVID-19 virus in Nepal.^11^ However, we couldn't retrieve any study done on the perception of common people about effectiveness of lockdown. In this study, it was found that some people were reusing the same mask. The hike in price and shortage may be the reason. To combat the shortage of mask during such crisis, FDA too recommended conservation strategies for use of mask by healthcare organizations and health-personnel only.^12^ FDA also recommended reuse of certain type of mask after decontamination.^13^

Our study showed majority of people being stressed 404 (72.8%) followed by 111 (20%) people with no stress, during lockdown period. A study done using Depression Anxiety Stress Scale (DASS-21) to analyze the psychological impact of COVID-19 in the 2530 members of the University of Valladolid, Spain showed that Moderate to extremely-severe scores of anxiety, depression, and stress was reported by 21.34%, 34.19% and 28.14% of the respondents, respectively.^14^ Another study done during this lockdown showed that there was increase in Google searches on boredom, loneliness, worry and sadness.^15^ A study done in 2009 during SARS outbreak showed that about 10% of the respondents had experienced high levels of posttraumatic stress (PTS) symptoms since the SARS outbreak.^16^ It is a normal human response to be stressed in a uncertain event like a pandemic. However, it is imperative to assess the anxiety and depression in the general population. We recommend use of standardized tool in order to assess these domains of mental health. Extensive study and management approach should be implemented during and after COVID-19 Pandemic to counter act these difficulties.

Majority of the respondents 353 (63.6%) were adversely affected by professional works or suffered economic loss in business. Supply of livelihood goods was hard for one fourth of our total respondents due to poor government support system in Nepal. A qualitative study done in India during this lockdown also reported that poor have lost the means of livelihood, the lockdown has the effect of making conditions worse for the poor.^17^ Practicing social distancing becomes too difficult for the poor in the absence of proper social security system and government support. More than 42% of the participants responded that they are using their time for study in personal development, online classes etc. and few have a fruitful and amazing family time. This shows that lockdown played positive impact on acceptance of online learning. A meta-analysis done in 2017 to see how effective online learning is for undergraduate medical education showed that there is no significant difference between online and offline teaching.^18^ Online study may increase after lockdown and be widely used by students and universities of medicine and other studies.

Majority of participants in our survey complied with the rules and regulations of the authority concerning lockdown, which is in consistent with the findings from Zhong et al that clearly illustrates people opinion to comply within lockdown norms and avoid crowds.^1^ Majority of respondent wanted prior concern to enhance testing followed by end of lockdown with the implementation of other strategies to control the infection. A significant number of people were responded skeptical and taken away from family members after returning home outside. This truly reflects the fear and stigma among them which is misleading^22^ about infection and perception of viral transmission due to inadequate tracing and testing status in Nepal. However, WHO has issued interim guidance on implementation to end lockdowns, which includes a step-wise approach that is adjusted according to local circumstances and prioritizes protecting vulnerable populations.^19^

Diarrhea related illness is still one of the common causes of mortality in a country like Nepal.^20,21^ The hand-washing practices and knowledge is sure to benefit in prevention of common gastrointestinal related infections in the days to come. But it is a dire need of time to reach as much as 11% of population in our survey who used just plain water as a method of hand sanitization. This number matters a lot when it comes to curtailing of the devastating pandemic like COVID-19. Further probing is necessary to find out if it was due to unavailability of soap, lack of knowledge or simple casualness. Also, the wonderful feeling and relatedness expressed by study subject infer the significance of survey like this which might represent a good information source for our policymakers to implement the further strategies to cope with such pandemics in days to come.

There are few limitations to this study. The sample was recruited using a non-randomized sampling procedure, limits the generalizability of present study findings. We assessed the variables during the early stage of lockdown and with time the knowledge and behavior of the population might have changed. The use of non-standardized questionnaire is another limitation of the study. There could have been duplication of the form fill-up which might skew the results. With all this limitations, we believe this study will act as a base for further robust studies in Nepalese population.

## CONCLUSIONS

Our study shows that lifestyle patterns, perception of viral infection and practices have been found to have improved due to the awareness rose during COVID-19 pandemic. However, majority want lockdown to be ended and other measures to be implemented to control the infection. Acceptance of online learning seems to be increased in medical education and other subjects. Many people suffered economic loss or lost their job during lockdown, which should be looked upon and solved. Risk of psychiatric problems may be seen as an after-effect of COVID-19 pandemic and lockdown; further studies and actions are needed to address it.

## References

[1] Zhong BL, Luo W, Li HM, Zhang QQ, Liu XG, Li WT, Li Y (2020). Knowledge, attitudes, and practices towards COVID-19 among Chinese residents during the rapid rise period of the COVID-19 outbreak: a quick online cross-sectional survey. Int J Biol Sci.

[2] Peeri Noah C, Shrestha Nistha, Rahman Md Siddikur, Zaki Rafdzah, Tan Zhengqi, Bibi Saana, Baghbanzadeh Mahdi, Aghamohammadi Nasrin, Zhang Wenyi, Haque Ubydul (2020). The SARS, MERS and novel coronavirus (COVID-19) epidemics, the newest and biggest global health threats: what lessons have we learned?. Int J Epidemiol.

[3] Centers for Disease Control and Prevention (2020). Clinical Care Guidance for Healthcare Professionals about Coronavirus (COVID-19).

[4] World Health Organization (2020). Director-General's remarks at the media briefing on 2019-nCoV on 11 February 2020.

[5] World Health Organization (2020). Novel coronavirus (2019-nCoV) situation report - 11.

[6] World Health Organization (2020). Rolling updates on coronavirus disease (COVID-19).

[7] World Health Organization (2020). Novel coronavirus (2019-nCoV) situation report - 153.

[8] Mao L, Xu J, Xu Z, Xia X, Li B, He J, Zhao P, Pan J, Zhang D (2020). A child with household transmitted COVID-19. BMC Infect Dis.

[9] World Health Organization (2020). Novel coronavirus (2019-nCoV) situation reports.

[10] Wikipedia (2020). COVID-19 pandemic in Nepal.

[11] Bhandary Shital (2020). Effectiveness of lockdown as COVID-19 intervention: official and computed cases in Nepal. Journal of Patan Academy of Health Sciences.

[12] US Food and Drug Administration (FDA) (2020). Shortages of Surgical Masks and Gowns during the COVID-19 Pandemic.

[13] US Food and Drug Administration (2020). FDA reissues EUAs revising which types of respirators can be decontaminated for use.

[14] Odriozola-González P, Planchuelo-Gómez Á, Irurtia MJ, de Luis-García R (2020). Psychological effects of the COVID-19 outbreak and lockdown among students and workers of a Spanish university. Psychiatry Res.

[15] Brodeur Abel, Clark Andrew, Flèche Sarah, Powdthavee Nattavudh (2020). Assessing the impact of the coronavirus lockdown on unhappiness, loneliness, and boredom using Google Trends.

[16] Wu P, Fang Y, Guan Z (2009). The psychological impact of the SARS epidemic on hospital employees in China: exposure, risk perception, and altruistic acceptance of risk. Can J Psychiatry.

[17] Thomas G (2020). Death in the time of coronavirus. Indian J Med Ethics.

[18] Pei L, Wu H (2019). Does online learning work better than offline learning in undergraduate medical education? A systematic review and meta-analysis. Med Educ Online.

[19] World Health Organization (2020). Considerations in adjusting public health and social measures in the context of COVID-19.

[20] Ghimire PR, Agho KE, Renzaho AM, Dibley M, Raynes-Gree-now C (2018). Association between health service use and diarrhoea management approach among caregivers of under-five children in Nepal. PloS one.

[21] Rai KR, Mukhiya RK, Thapa S, Rai G, Sabina KC, Thapa PM, Shrestha P, Rai SK (2019). Diarrheal disease outbreak in Gaidatar village of Rautahat District, Nepal. BMC research notes.

[22] Ren S-Y, Gao R-D, Chen Y-L (2020). Fear can be more harmful than the severe acute respiratory syndrome coronavirus 2 in controlling the corona virus disease 2019 epidemic. World J Clin Cases.

